# The Impact of Parents' Categorization of Their Own Weight and Their Child's Weight on Healthy Lifestyle Promoting Beliefs and Practices

**DOI:** 10.1155/2015/307381

**Published:** 2015-03-16

**Authors:** Allison C. Sylvetsky-Meni, Scott E. Gillepsie, Trisha Hardy, Jean A. Welsh

**Affiliations:** ^1^Department of Exercise and Nutrition Sciences, The George Washington University, 950 New Hampshire Avenue NW, Washington, DC 20052, USA; ^2^Graduate Division of Biological and Biomedical Sciences, Laney Graduate School, Emory University, 201 Dowman Drive, Atlanta, GA 30322, USA; ^3^Department of Pediatrics, Emory School of Medicine, 1760 Haygood Drive NE, Atlanta, GA 30322, USA; ^4^Child Wellness Department, Children's Healthcare of Atlanta, 1577 NE Expressway, Atlanta, GA 30329, USA

## Abstract

*Objective*. To evaluate parents' beliefs and practices related to childhood obesity and determine if these are influenced by parent's perception of their own weight or their child's weight. *Methods*. Parents of obese (*n* = 689) or normal weight (*n* = 1122) children 4–15 years in Georgia, USA, were randomly selected to complete a telephone survey. Frequency of child obesity-related perceptions, beliefs, and practices were assessed, stratified by parent-perceived self-weight and child weight status, and compared using Chi-squared tests and multivariate logistic regression. *Results*. Most parents, regardless of perceived child weight, agreed that child overweight/obesity can cause serious illness (95%) but only one-half believed it was a problem in Georgia. Many (42.4%) failed to recognize obesity in their own children. More parents who perceived their child as overweight versus normal weight reported concern about their child's diet and activity and indicated readiness for lifestyle change. Parents' perception of their own weight had little additional impact. *Conclusions*. While awareness of child overweight as a modifiable health risk is high, many parents fail to recognize it in their own families and communities, reducing the likelihood of positive lifestyle change. Additional efforts to help parents understand their role in facilitating behavior change and to assist them in identifying at-risk children are required.

## 1. Introduction

Since the 1970s, the prevalence of childhood obesity has nearly tripled in the United States [1], with the current rate approaching 20%. Obesity in childhood is associated with increased risk of chronic diseases, including type 2 diabetes and nonalcoholic fatty liver disease, as well as depression and other psychosocial conditions [2]. In addition, children who begin their lives overweight or obese are at heightened risk of adult obesity and related comorbidities [3].

Having an overweight or obese parent is known to greatly increase a child's risk of obesity. While research suggests that a proportion of this risk can be attributed to genetics, much is believed to result from environmental factors and is therefore modifiable [4, 5]. Increased fruit and vegetable consumption, decreased sugar-sweetened beverage consumption, increased physical activity, and decreased use of media and other sedentary activities have been recommended as promising strategies for decreasing obesity risk [6]. Little is known about how to best modify diet and physical activity behaviors that promote a healthy weight. Parental involvement is believed to be a critical component of successful weight management practices in children [7–10]; yet, it is unclear as to why some parents practice behaviors that support the development of a healthy lifestyle, while others do not.

Previous research suggests that how parents perceive the weight status of their children affects the extent to which they support positive health and activity behaviors in the family [11–13]. Parents who correctly recognize that their child is overweight more often indicate concern [14] and report intentions to positively modify family lifestyle behaviors [15] compared to parents who incorrectly categorize their overweight or obese children as being healthy weight. This suggests that parents who perceive their children as normal weight, correctly or not, may be doing less to promote healthy diet and activity practices in the family. This is important as behavior patterns established in childhood are associated with future obesity and chronic disease risk [3]. The purpose of this study was to evaluate parental beliefs about childhood obesity and about their child's diet and physical activity practices. We also aimed to determine if these beliefs and practices are modified by parent's perception of their own weight and/or the weight of their child.

## 2. Methods

### 2.1. Sample

Data were collected from November 2012 to February of 2013, as part of a telephone survey designed to assess the impact of a marketing campaign sponsored by Children's Healthcare of Atlanta. List-assisted, random-digit dialing was used to select households throughout Georgia, USA. Parents or caregivers (referred to hereafter as parents) living in the selected households were called and invited to participate if they had ≥ 1 child between the ages of 4 and 15 who was either normal weight (defined as 5th = BMI percentile < 85th percentile for their age and sex per the 2000 Centers for Disease Control and Prevention (CDC) Growth Charts) or obese (BMI ≥ 95th percentile) [16]. Estimates of BMI were derived using parent-reported values for child height and weight. Parents were excluded from participating if they did not speak either English or Spanish or if they were not a resident of Georgia. Up to six attempts were made to reach parents before replacing them with a randomly selected alternate. If households met the criteria and parents agreed to participate, the interview proceeded immediately. The study protocol was reviewed by the Institutional Review Board at Children's Healthcare of Atlanta and determined to be exempt from federal regulations regarding human subjects research.

Quota sampling was used to ensure recruitment of a large and approximately equal number of parents of children who were obese and parents of children who were normal weight. Respondents with two or more children meeting the survey criteria were asked to complete the interview based on a single, randomly selected child. A total of 1811 parents agreed to participate and fully completed the interview; 1122 were parents of normal weight children and 689 were parents of obese children, based on standard CDC guidelines.

### 2.2. Measures

Demographic information collected included location of residence (grouped by zip code as metropolitan Atlanta, other urban, and other), family annual income level (<$50,000 or ≥$50,000), education level (some college or less versus college graduate), sex, race/ethnicity (non-Hispanic white; non-Hispanic black; Hispanic; or other), and parental self-reported weight category (normal weight versus overweight or very overweight/obese (overweight)). Information collected from parents regarding their child included sex, age (years), estimated height (feet and inches), and weight (pounds). These data were used to derive the child's weight status (derived weight status) in order to determine study eligibility. Parents were also asked to report their perception of their child's weight status (perceived weight status) as either underweight or normal weight (normal weight) or overweight or extremely overweight/obese (overweight).

Data were collected using a questionnaire designed to evaluate parental attitudes and practices regarding childhood obesity, as well as their child's diet and physical activity. Responses were measured using a Likert scale, with options ranging from 1 = strongly disagree to 10 = strongly agree. Interviewers read and recorded parent responses for each of the 18 statements relating to child obesity (3 items), their role as parents (3 items), their child's weight (2 items), their child's diet (7 items), and their child's physical activity (3 items). Examples of statements included “I try to educate my child about healthy eating choices,” “it costs too much to eat healthy,” “technology causes my child to spend less time physically active than I would prefer,” “how concerned, if at all, are you about what your child eats,” and “as long as my child is happy, it does not matter what he or she weighs” (see Table 2 for a listing of all statements).

### 2.3. Statistical Analysis

Data analyses were performed using SAS 9.3 (Cary, NC), and statistical significance was evaluated at the 0.05 level. All demographic characteristics were summarized using frequency counts and percentages. Percentages of agreement (score ≥ 7 out of 10) were calculated for each questionnaire item and compared across parent self-reported weight status (normal weight versus overweight) and parent reported child weight status (normal weight versus overweight) using Chi-square tests of independence or Fisher's exact tests. Multiple logistic regression was employed to calculate adjusted odds ratios and 95% CI for each question, while controlling for potentially confounding variables including parent sex, race/ethnicity, income, parent education, geographic residence, child sex, and child age. Using parents who perceived their child as normal weight as the reference group, adjusted odds ratios compared their responses to those of parents who perceived their child as overweight. Among the subsample including only parents of children whom they perceived to be overweight, responses were also assessed based on the parents' self-reported weight status (normal weight versus overweight).

## 3. Results

The majority of parents studied were female (73.1%) and non-Hispanic white (65.7%). Half (50.8%) of those surveyed had a college education or higher, and 58.9% reported an annual household income ≥ $50,000 (not shown). Parent demographic characteristics stratified by their perception of their child's weight status are shown in Table 1. There were no statistically significant differences in the two parent groups with regard to parent sex, education level, household location, or child sex. Parents who perceived their child as overweight were more likely to be non-Hispanic black (31.2% versus 23.6%) or Hispanic (6.1% versus 4.3%) (*P* = 0.003) and to be of low income (50.6% versus 38.8%; *P* < 0.001) than parents of children perceived as normal weight.

Comparisons between child weight status as perceived by their parent and BMI-derived child weight status (using standard BMI cutoffs) demonstrated that, of the children perceived by their parents to be normal weight, 72.8% met the BMI-derived criteria for normal weight, while 83.6% of those perceived by parents to be overweight met the BMI-derived criteria of overweight or obese (Table 1). Of those children classified as normal weight using their BMI-derived weight status, 95% were correctly perceived as normal weight by parents; yet of those children classified overweight/obese using BMI, only 42.2% were correctly perceived as overweight by their parents.

### 3.1. Parental Beliefs and Practices Related to Childhood Obesity

Parental beliefs and practices related to child obesity, stratified by their perception of their child's weight, are summarized in Table 2. Among those parents who perceived their child as overweight, responses were also compared based on the parents' self-categorization of their own weight status. Awareness that child obesity can cause serious illness was high among all parents (94.8%), though less than half believed child obesity to be a serious problem in their state (44.5%). Parents who perceived their child as overweight were more likely than those who perceived their child as normal weight to report that child obesity was a serious problem (53.5% versus 42.3%, *P* < 0.001). Few parents believed that little can be done because child obesity runs in families; however, more parents who perceived their child as overweight versus normal weight agreed with this statement (12.1% and 8.4%, resp., *P* = 0.034).

### 3.2. Parents as Role Models

Nearly all parents (94.1% overall) recognized the importance of being a healthy role model for their children irrespective of their perceptions of their child's weight. Few parents (5.3% overall), regardless of their perception of their child's weight status, agreed with the statement that there is little that parents need to do for overweight children as they will grow out of it.

### 3.3. Parental Perceptions of Child's Weight

As expected, parents who perceived their child as overweight were significantly more likely to be concerned about their child's weight (73.0% versus 14.4%, *P* < 0.001) (Table 2) than parents who perceived their child as normal weight. Parents who perceived their child as overweight were also more likely to report readiness to make nutrition and physical activity changes (58.6% versus 26.4%, *P* < 0.001).

### 3.4. Parental Beliefs and Practices Related to Their Child's Diet

Nearly all parents, regardless of their perception of their child's weight, reported trying to teach their children about healthy eating (90.3%). Parents who perceived their child as overweight versus normal weight were more likely to be concerned about their family's eating habits, 85.3% versus 55.8% (*P* < 0.001), and to be actively working to change them for the better, 78.5% versus 70.5% (*P* = 0.003). These parents were less likely to report that they (parents of perceived overweight children) can provide healthy meals even when they are busy (72.4% versus 79.6%, *P* = 0.005). Approximately half of all parents agreed that it takes more time to eat healthy (50.7%), and roughly one-third indicated that it costs too much to eat healthy (35.3%), with no differences between parents by the perceived weight status of their child.

### 3.5. Parental Beliefs and Practices Related to Their Child's Physical Activity

As with diet, parents who perceived their child as overweight more frequently reported concern about how much physical activity their child gets (83.1% versus 55.2%, *P* < 0.001) compared to parents who perceived their child as normal weight. Similarly, parents who perceived their child as overweight frequently reported that technology caused their children to spend less time being physically active than they preferred (54.3% versus 35.8%, *P* < 0.001).

### 3.6. Influence of Parental Self-Reported Weight Status on Child Obesity Related Beliefs and Practices

Among the subsample including only parents who perceived their child as overweight, few significant differences in responses were observed based on self-reported parental weight status. However, parents self-perceived as overweight were less likely to agree that they could provide healthy meals for their child even when busy (67.3% versus 81.6%, *P* = 0.004) and were more likely to believe that it takes more time and effort to eat healthy (59.2% versus 43.2%, *P* = 0.004).

Adjusted odds ratios (AORs) for each of the 18 questionnaire items are presented in Table 3. After controlling for demographic characteristics including parent sex, race/ethnicity, income, parent education, geographic residence, child sex, and child age, response patterns across the various parent subgroups outline above remained largely the same. Two of the weaker associations involving the questions, “little can be done for overweight children because weight problems run in families” and “it costs too much to eat healthy,” were no longer statistically significant after adjusting for parent and child characteristics.

## 4. Discussion

In the present study, we assessed parental beliefs and practices regarding child obesity and their child's diet and physical activity-related behaviors. We then compared finding by parents' perception of their own weight and that of their child. Similar to the results of earlier studies [11, 12, 17], we found that many parents of obese children incorrectly perceived these children as normal weight. Since parents who perceive their obese children as normal weight are less likely to be motivated to make healthy lifestyle behavior changes [15], this highlights the need for increased effort to ensure that children's weight status is regularly and accurately assessed and effectively communicated to parents.

Our results demonstrate that nearly all parents recognize that obesity can cause serious illness and most, regardless of their perception of their own weight status or that of their child, reported that they were trying to educate their children about healthy eating choices and were working to improve their family's diet. Despite this widespread awareness that obesity could lead to serious health complications, our analysis revealed important differences in parents' beliefs and practices depending on how they perceived their child's weight.

Parents who perceived their child as overweight were more likely to view their child's diet, physical activity patterns, and risk of obesity as a health threat and childhood obesity as a serious problem. Despite increased perception of a health threat among parents who perceived their child as overweight, these parents were also more likely to report that little can be done because obesity runs in families and that their child's use of technology prevented them from being physically active. This suggests that education about obesity prevention and assistance to overcome barriers, rather than simply informing parents about risk, are most needed to support their healthy behavior change efforts.

While healthy weight promoting beliefs and practices differed significantly by parental perception of their child's weight, parental self-report of their own weight category had little additional impact. One difference was our finding that parents self-categorized as overweight less frequently indicated being able to provide healthy meals when busy and often reported that it takes more time and effort to eat healthy, similar to barriers described in prior studies [18, 19]. This suggests a potentially important discrepancy between parental concern, which was high among parents who perceived their child as overweight regardless of their own weight status, and parental ability to take action. While the basis for this discrepancy requires further exploration, one possible explanation may be that parents self-categorized as overweight lack the confidence to promote lifestyle modification [20–22], perhaps because these parents have been unsuccessful in controlling their own weight. Thus, programs devised to increase parental self-efficacy [9, 23] may be most effective in promoting health behavior change among overweight and obese parents.

Our study builds upon the existing obesity literature in examining the impact of parental perception of both their own weight status and their child's weight category on their obesity-related beliefs and practices. Though the generalizability of our results is limited by the recruitment of a convenience sample of parents, the use of random sampling to identify parents throughout the state helped to increase the geographic representativeness of the sample. While reliance on parent-reported child height and weight as measure of true weight status for purposes of study enrollment may have resulted in some misclassification, inclusion of only children determined to be either normal weight or obese and excluding those in between (determined to be overweight) are expected to have minimized such misclassification. Oversampling of parents with children identified as obese using parent reported height and weight ensured a large enough sample to allow for comparisons with parents of normal weight children.

## 5. Conclusions

The results of our study demonstrate that awareness of child obesity as a modifiable but serious health risk is high among parents, though many do not recognize its relevance in their own lives. This may result in a failure to promote positive health behaviors in their own family and failure to support childhood obesity prevention efforts in their communities. Further effort is needed to help parents understand the importance of their role in promoting positive lifestyle behaviors and to assist parents in identifying those children whose weight status places them at increased risk, while providing additional counseling and support necessary to make behavior changes.

## Figures and Tables

**Table 1:** Characteristics of parents stratified by perceived child's weight status (*n* = 1811) and by parents' self-reported weight status only among parents who perceived their child as overweight.

Characteristic	All parents (*n* = 1811)		Parents of overweight^1^ child (*n* = 348)	
	Child normal weight^1^	Child overweight^1^	Normal weight^1^parent	Overweight^1^ parent
	*N* = 1463	*N* = 348	*N* = 125	*N* = 223
Parent/household				
Sex of parent				
Female	72.3%	76.4%	72.0%	78.9%
Male	27.7%	23.6%	28.0%	21.1%
Race/ethnicity				
Non-Hispanic white	67.7%	57.2%^**^	62.6%	54.3%
Non-Hispanic black	23.6%	31.2%	26.0%	34.1%
Hispanic	4.3%	6.1%	5.7%	6.3%
Other	4.4%	5.5%	5.7%	5.4%
Household income^1^				
<$50,000	38.8%	50.6%^***^	44.5%	54.0%
≥$50,000	61.2%	49.4%	55.5%	46.0%
Education				
Some college or less	48.2%	53.5%	55.2%	52.5%
College graduate	51.8%	46.5%	44.8%	47.5%
Household location				
Metro	43.7%	47.7%	46.4%	48.4%^*^
Other urban	33.8%	31.0%	25.6%	34.1%
Other	22.5%	21.3%	28.0%	17.5%
Child				
Weight status (per BMI)				
Normal weight	72.8%	16.4%^***^	16.8%	16.1%
Obese	27.2%	83.6%	83.2%	83.9%
Sex of child				
Female	48.1%	49.1%	40.0%	54.3%^*^
Male	51.9%	50.9%	60.0%	45.7%
Age of child				
≤11 years	59.5%	50.0%^**^	55.2%	47.1%
>11 years	40.5%	50.0%	44.8%	52.9%

^*^ *P* < 0.05; ^**^ *P* < 0.01; ^***^ *P* < 0.001.

Two-sided level of significance in relation to their normal weight comparator was tested using Chi-square tests of independence or Fisher's exact tests.

^1^Weight status of parents and children is that as perceived by the parents.

**Table 2:** Proportion of parents agreeing with statements related to childhood obesity, diet, and physical activity stratified by child and parent weight status (as perceived by parents).

Survey item	All parents		Parents of overweight^1^ child	
	Child normal weight^1^	Child overweight^1^	Normal weight^1^ parent	Overweight^1^ parent
	*N* = 1463	*N* = 348	*N* = 125	*N* = 223
*Childhood obesity-general *				
Being overweight or obese as a child can cause serious illnesses	94.7%	95.4%	92.8%	96.9%
Childhood obesity is a serious problem in Georgia	42.3%	53.5%^***^	49.6%	55.6%
Little can be done for overweight children because weight problems run in families	8.4%	12.1%^*^	11.2%	12.6%
*Parents as role models *				
It is important for me to eat well and be active because I am a role model for my child	94.3%	93.4%	94.4%	92.8%
Parents do not need to do anything about child overweight as they will grow out of it	4.9%	7.2%	8.0%	6.7%
*Child's weight *				
I am concerned with my child's weight	14.4%	73.0%^***^	69.6%	74.9%
I am ready to address my child's weight	26.4%	58.6%^***^	58.4%	58.7%
As long as my child is happy, his/her weight does not matter	7.5%	10.3%	11.2%	9.9%
*Child's diet *				
I try to teach my child about healthy eating choices	90.1%	91.1%	93.6%	89.6%
I am concerned with what my child eats	55.8%	85.3%^***^	84.0%	86.1%
I am trying to change my family's eating habits for the better	70.5%	78.5%^**^	76.8%	70.4%
My friends and family think it's important that children eat a healthy diet	87.3%	82.1%^*^	81.6%	82.4%
I can provide healthy meals for my child even when busy	79.6%	72.4%^**^	81.6%	67.3%^**^
It takes more time and effort to eat healthy	50.0%	53.5%	43.2%	59.2%^**^
It costs too much to eat healthy	34.2%	39.9%^*^	34.4%	43.1%
*Child's physical activity *				
I am concerned with how much activity or exercise my child gets	55.2%	83.1%^***^	82.4%	83.4%
I am ready to make nutrition and activity change today	26.4%	58.6%^***^	58.4%	58.7%
Technology causes my child to spend less time in physical activity than I would prefer	35.8%	54.3%^***^	54.4%	54.3%

^*^ *P* < 0.05; ^**^ *P* < 0.01; ^***^ *P* < 0.001.

Two-sided level of significance in relation to their normal weight comparator was assessed using Chi-square tests of independence or Fisher's exact tests.

^1^Weight status of parents and children is that as perceived by the parents.

**Table 3:** Multivariable adjusted odds ratios (AORs) of parental agreement survey items related to childhood obesity, diet, and physical activity (*n* = 1811).

Survey item	All parents of overweight^1^ versus normal weight^1^ child	Overweight^1^ versus normal weight^1^parents of overweight^1^ children
	AOR (95% CI)	AOR (95% CI)
*Childhood obesity-general *		
Being overweight or obese as a child can cause serious illnesses	1.09 (0.62–1.93)	3.11 (0.99–9.73)
Childhood obesity is a serious problem in Georgia	1.52 (1.19–1.94)^***^	1.43 (0.89–2.31)
Little can be done for overweight children because weight problems run in families	1.28 (0.87–1.91)	0.98 (0.43–2.21)
*Parents as role models *		
It is important for me to eat well and be active because I am a role model for my child	0.96 (0.58–1.60)	0.57 (0.19–1.67)
Parents don't need to do anything about child overweight as they will grow out of it	1.42 (0.87–2.31)	0.77 (0.32–1.87)
*Child's weight *		
I am concerned with my child's weight	17.06 (12.65–23.01)^***^	1.18 (0.69–2.01)
I am ready to address my child's weight	3.76 (2.91–4.86)^***^	0.88 (0.54–1.42)
As long as my child is happy, his/her weight doesn't matter	1.36 (0.90–2.07)	0.79 (0.37–1.69)
*Child's diet *		
I try to teach my child about healthy eating choices	1.16 (0.76–1.78)	0.54 (0.22–1.37)
I am concerned with what my child eats	4.32 (3.12–5.98)^***^	1.10 (0.56–2.14)
I am trying to change my family's eating habits for the better	1.49 (1.11–2.00)^**^	1.11 (0.62–1.97)
My friends and family think it's important that children eat a healthy diet	0.69 (0.50–0.97)^*^	0.97 (0.52–1.81)
I can provide healthy meals for my child even when busy	0.70 (0.53–0.93)^*^	0.47 (0.27–0.85)^*^
It takes more time and effort to eat healthy	1.15 (0.90–1.47)	1.83 (1.13–2.96)^*^
It costs too much to eat healthy	1.13 (0.87–1.46)	1.58 (0.96–2.61)
*Child's physical activity *		
I am concerned with how much activity or exercise my child gets	3.66 (2.69–5.00)^***^	0.95 (0.51–1.77)
I am ready to make nutrition and activity change today	3.76 (2.91–4.86)^***^	0.88 (0.54–1.42)
Technology causes my child to spend less time in physical activity than I would prefer	2.01 (1.56–2.58)^***^	1.01 (0.62–1.63)

^*^ *P* < 0.05; ^**^ *P* < 0.01; ^***^ *P* < 0.001 (two-sided level of significance in relation to their normal weight comparator).

^†^Odds ratios were calculated using logistic regression and were adjusted for parent gender, race/ethnicity, income, parent education, geographic residence, child gender, and child age.

^1^Weight status of parents and children is that as perceived by the parents.
